# Generation of an Ovomucoid-Immune scFv Library for the Development of Novel Immunoassays in Hen’s Egg Detection

**DOI:** 10.3390/foods12203831

**Published:** 2023-10-19

**Authors:** Santiago Rodríguez, Aina García-García, Eduardo Garcia-Calvo, Vanesa Esteban, Carlos Pastor-Vargas, Araceli Díaz-Perales, Teresa García, Rosario Martín

**Affiliations:** 1 Departamento de Nutrición y Ciencia de los Alimentos, Facultad de Veterinaria, Universidad Complutense de Madrid (UCM), 28040 Madrid, Spain; santro03@ucm.es (S.R.); edugar01@ucm.es (E.G.-C.); tgarcia@ucm.es (T.G.); rmartins@ucm.es (R.M.); 2 Departamento de Alergia e Inmunología, IIS-Fundación Jiménez Díaz, Universidad Autónoma de Madrid (UAM), 28040 Madrid, Spain; vesteban@fjd.es; 3 Departamento de Bioquímica y Biología Molecular, Facultad de Ciencias Químicas, Universidad Complutense de Madrid, 28040 Madrid, Spain; cpasto01@ucm.es; 4 Centro de Biotecnología Y Genómica de Plantas, Universidad Politécnica de Madrid-Instituto Nacional de Investigación y Tecnología Agraria y Alimentaria (CBGP, UPM-INIA), 28223 Madrid, Spain; araceli.diaz@upm.es

**Keywords:** ovomucoid, phage display, immune library, recombinant antibody, scFv, ELISA, egg detection

## Abstract

Hen’s egg allergy is the second most common food allergy among infants and young children. The possible presence of undeclared eggs in foods poses a significant risk to sensitized individuals. Therefore, reliable egg allergen detection methods are needed to ensure compliance with food labeling and improve consumer protection. This work describes for the first time the application of phage display technology for the generation of a recombinant antibody aimed at the specific detection of hen’s ovomucoid. First, a single-chain variable fragment (scFv) library was constructed from mRNA isolated from the spleen of a rabbit immunized with ovomucoid. After rounds of biopanning, four binding clones were isolated and characterized. Based on the best ovomucoid-binding candidate SR-G1, an indirect phage enzyme-linked immunosorbent assay (phage-ELISA) was developed, reaching limits of detection and quantitation of 43 and 79 ng/mL of ovomucoid, respectively. The developed ELISA was applied to the analysis of a wide variety of food products, obtaining a good correlation with a commercial egg detection assay used as a reference. Finally, in silico modeling of the antigen-antibody complex revealed that the main interactions most likely occur between the scFv heavy chain and the ovomucoid domain-III, the most immunogenic region of this allergen.

## 1. Introduction

Egg allergy is one of the most prevalent food allergies in infants and young children, ranking second only to cow’s milk allergy [1]. A rise in its incidence has been reported and is currently estimated to affect approximately 0.5–2.5% of children worldwide [2,3]. Allergic reactions to eggs can range from mild skin symptoms and rhinitis to more severe conditions such as asthma, chronic gastroenteropathy, and even anaphylaxis, posing a substantial threat to sensitized individuals [4,5]. Most of the allergenic egg’s proteins are found in egg white, with ovomucoid (Gal d 1) standing out as the dominant allergen [1,6] due to its unique characteristics, such as heat stability, resistance to proteolytic digestion [7,8] and its strong allergenicity compared to other egg allergens [9]. Ovomucoid, which represents 11% of the egg white proteins, is a 28 kDa protease inhibitor composed of three interconnected tandem domains (DI, DII, and DIII), with the third domain showing significantly higher reactivity to IgG and IgE [10]. These attributes make ovomucoid an interesting target for allergen detection in the fields of food safety and egg allergy research.

To protect allergic consumers, the most effective measure is to avoid the consumption of the offending food. For this reason, the European Union has adopted labeling requirements for 14 allergenic foods, including eggs [11]. However, egg traces may unexpectedly be present in foodstuffs where the egg is not labeled as an ingredient. Sources of this food contamination could include formerly contaminated raw materials, inadequate cleaning along the food processing line, or other sources of cross-contact. Nevertheless, enforcement of these regulations depends on the availability of sensitive and specific analytical methods that allow verification of the accuracy of the labeling information.

Immunological techniques, in particular ELISA methods, have become routine methods for the detection and quantification of egg allergens in commercial products due to their sensitivity, cost-effectiveness, time-saving, and ease of use [12]. The performance of ELISA assays relies on the availability of highly sensitive and specific antibodies against the target antigen. Traditional methods to obtain specific antibodies involve the use of antisera from immunized animals or hybridoma antibody production. However, these approaches suffer from drawbacks such as a limited supply of antisera with batch-to-batch variations or the technical complexity of hybridoma cell line production. Considering these challenges, recombinant antibodies are emerging as a promising alternative to hybridoma technology for the generation of monoclonal antibodies. Recombinant antibody production offers several advantages, such as faster development time, lower production costs, easier scalability and continuous supply with batch-to-batch consistency, increased reproducibility, and the possibility of implementing animal-free procedures [13].

In this context, phage display technology has proven to be one of the most powerful tools for selecting high-affinity probes, making it a valuable in vitro tool for generating recombinant antibodies using a direct molecular evolution process [14]. In this methodology, antibody fragments are expressed as fusion proteins on the surface of filamentous bacteriophages, thereby linking phage phenotype and genotype and allowing immortalization of the monoclonal antibody genes. Moreover, it offers the possibility of tailoring the antibodies to suit different applications, such as improving stability or binding affinity or fusing them to a wide variety of reporter genes [15]. Among the different formats of antibody fragments that could be correctly displayed on filamentous phage, the single-chain variable fragments (scFv), consisting of a variable region of a heavy (V_H_) and a light chain (V_L_) linked by a flexible linker, have demonstrated their suitability for the detection of allergens in foods with appropriate affinity and specificity [16,17,18,19].

Rabbits are a prominent and well-established choice for generating both polyclonal and monoclonal antibodies, owing to their unique characteristics. They are known for their ability to produce large quantities of high-affinity antibodies, often exhibiting picomolar equilibrium dissociation constants [20]. Furthermore, in contrast to rodents that are evolutionarily closer to humans, rabbit-derived antibodies possess the potential to recognize epitopes that are poorly immunogenic in mice and humans [21]. For these reasons, both naïve and immune phage libraries are frequently constructed from antibody genes collected from bone marrow, spleen, and other rabbit samples [20,22].

Phage display technology has been successfully applied for the detection of different allergens and pathogens related to food safety [17,23,24,25,26]. However, studies concerning the generation of specific recombinant antibodies against the ovomucoid egg protein by phage display have not been described to date. The present study addresses this unexplored issue via the construction of a phage library displaying monoclonal scFvs obtained from an ovomucoid-immunized rabbit. The objective of this approach was the development of an indirect phage-ELISA based on the best-performing scFv candidate for the detection of hen’s egg in commercial food products.

## 2. Materials and Methods

### 2.1. Immunization

A healthy New Zealand white rabbit was immunized with 550 µL containing 100 µg of ovomucoid in complete Freund’s adjuvant (Difco™-Thermo Fisher©, Waltham, MA, USA, #ref DF0638-60-7). The rabbit was injected intramuscularly once a week for a 6-week period, and the spleen was collected seven days later to construct the phage display library. The protocol for research on animals was approved by Animal Core Facility Health Research Institute (Fundación Jiménez Díaz) and Subdirecciȯn General de Producción Agroalimentaria y Bienestar Animal (Comunidad de Madrid) (Approval Number: PROEX 132/19).

### 2.2. Materials and Bacterial Strains

*Escherichia coli* XL1-Blue strain (*recA1 endA1 gyrA96 thi-1 hsdR17 supE44 relA1 lac* [F’ *proAB lacI^q^ZΔM15 Tn10* (*Tet^r^*)]) obtained from Agilent©, Santa Clara, CA, USA, (#ref 200150) was employed for library construction into pComb3X vector and phage-displayed scFvs production. Electrocompetent *E. coli* cells were produced in-house by culturing in a super-broth medium (SB: 30 g/L tryptone, 20 g/L yeast extract, 10 g/L MOPS pH 7). Subsequent centrifugation cycles were performed, followed by resuspension in 10% (*v*/*v*) glycerol (PanReac AppliChem©, Monza, Lombardy, Italy, CAS: 56-81-5) as described by Barbas et al. [27]. Following electroporation (2.50 kV pulse), cells were cultured in SOC medium (Invitrogen™-Thermo Fisher©, #ref 15544-034). For DNA extraction prior to sequencing, bacteria were grown in Luria Broth (LB: 10 g/L tryptone, 5 g/L yeast extract, 10 g/L NaCl). Agar plates were prepared with 15 g/L agar concentration.

### 2.3. Preparation of Antigen and Food Protein Extracts

Gal d 1 (trypsin inhibitor from chicken egg white from Sigma©, Burlington, MA, USA, #ref SLCD5629) was used as a reference material for ovomucoid detection in foods. Ovalbumin (Sigma©, #ref 47H0966) and lysozyme (Sigma©, #ref L6876) were employed to assess the specificity of the obtained antibodies against other egg allergens.

A diverse array of plant and animal species (Table 1) and eggs from different poultry species (chicken, quail, duck, goose, and ostrich) included in the specificity tests, as well as the 23 analyzed commercial food products, were procured from retail establishments and local markets in Madrid (Spain). After finely grinding the samples (50 g) in a food processor, they were placed into screw-capped vials and stored at −20 °C until further protein extraction prior to analysis.

Protein extracts were prepared by mixing 100 mg of the sample with 1 mL of PBS 1M (pH 7.4) in a microcentrifuge tube. The resulting mixture was vortexed for 1 min, followed by an incubation at 60 °C for 30 min with continuous stirring at 950 rpm. Then, the slurry was centrifuged at 10,000× *g* for 15 min at 4 °C. The resulting supernatants were carefully preserved at −20 °C until further analysis.

### 2.4. DNA Isolation, Quantification, and Cloning

All DNA products used for library construction were isolated by gel electrophoresis using UltraPure™Low melting point agarose (Thermo-Fisher©, #ref 16520050) and purified with NucleoSpin^®^ gel DNA clean-up columns (Machery-Nagel©, Allentown, PA, USA, #ref 740609). DNA was quantified in a Qubit^®^ Fluorometer (Invitrogen), and its quality was measured with a NanoDrop ND-1000 spectrophotometer (NanoDrop Technologies Inc., Montchanin, DE, USA). Restriction enzymes *Sfi*I (#ref R0123), *Nco*I (#ref R0193)*, Not*I (#ref R0189), and T4 DNA ligase (#ref M0202) used for DNA cloning were purchased from New England Biolabs© (Ipswich, MA, USA).

### 2.5. Construction of the Recombinant scFv Immune Library

Total RNA was extracted from the spleen tissue of the ovomucoid-immunized rabbit by TRIzol reagent (Invitrogen, #ref 10296010) according to the manufacturer’s instructions, and its purity determined from the absorbance ratio at OD260/OD280 measured with a NanoDrop. After genomic DNA treatment with ezDNase (Invitrogen, #ref 11766051), cDNA was generated using the SuperScript IV First-Strand Synthesis system (Invitrogen, #ref 16397225). The variable regions of heavy-chain (VH), κ light-chain (VLκ), and λ light-chain (VL_λ_) immunoglobulin genes were amplified by conducting more than 100 independent PCR reactions covering all possible combinations of the different set of primers described in Table S1. All primers were synthesized by Eurofins genomics© (Luxemburg). The 5′-primers for VL and 3′-primers for VH amplification were designed to include a SfiI restriction site, whereas the 5′-primers for VH and 3′-primers for VL incorporated part of the linker (SSGGGGSGGGGGGSSRSS). The first round of PCR reactions, conducted to separately amplify VL and VH chains, consisted of a 100 μL reaction of AmpliTaqGold^®^ (Thermo Fisher©, ref #4311806) polymerase containing 4 μL of cDNA, 20 pmol of both the upstream and downstream primers and 8 µL of 2.5 mM dNTPs (Thermo Fisher©, #ref AB0196). For VL amplification, the reaction conditions were as follows: initial denaturation at 95 °C for 10 min, followed by 35 cycles of denaturation at 94 °C for 15 s, annealing at 54 °C for 30 s, extension at 72 °C for 90 s, and a final extension at 72 °C for 10 min. For VH amplification, the reaction conditions were the same as those specified for VL but for an annealing temperature of 66 °C. The resulting PCR products were combined to obtain two independent VL and VH DNA pools. The VL-linker-VH sequences were obtained from 100 overlap extension PCR (SOE-PCR) reactions performed in the same conditions as the first round of PCRs but using 50 ng of each DNA pool and 10 pmol of the upstream (5′*Sfi*I-VL) and downstream (3′*Sfi*I-VH) primers (Table S1). The SOE-PCR conditions consisted of an initial denaturation at 95 °C for 10 min, followed by 25 cycles of denaturation at 94 °C for 15 s, annealing at 60 °C for 30 s, extension at 72 °C for 120 s, and a final extension at 72 °C for 10 min. Both the resulting DNA fragments and the pComb3X vector were digested with *Sfi*I at 50 °C for 2 h. Subsequently, the digested scFv fragments and vector were gel-purified and ligated in a 3:1 (insert:vector) molar ratio using T4 DNA ligase at 16 °C for 16 h. From this point forward, the library construction process followed the protocol described by Garcia-Calvo et al. [24]. Briefly, the resulting ligation underwent ethanol precipitation, followed by resuspension in 20 µL of water, and was transformed into electrocompetent *E. coli* XL1-Blue (2500 V). After SOC recovery, the repertoire size of the library was calculated by platting ten-fold serial dilutions. Finally, the culture was infected with VSCM13 helper phage, and the phage-scFv library was isolated from the culture supernatant by PEG-NaCl precipitation.

Twenty individual library-transformed colonies were randomly selected and grown overnight in 5 mL of LB medium supplemented with 100 µg/mL of carbenicillin. Plasmid DNA was isolated from the pellet using a mini-prep kit (GenElute™ plasmid miniprep kit from Sigma©, ref #PLN70). The phagemids were sequenced using the Sanger method (Eurofins genomics©) using the primers ompAseq and g-back (Table S1). Sequences were analyzed and visualized using Snapgene software (Dotmatics^®^, Boston, MA, USA).

### 2.6. Biopanning of the scFv Library against Ovomucoid and Egg White Extract

Four wells from two independent 96-well immunoplates were, respectively, coated for 1 h at 37 °C with 100 µL of Gal d 1 (10 µg/mL) and boiled egg white protein extract (1:100 dilution) in PBS (137 mM NaCl, 2.7 KCl, 10 mM Na_2_HPO_4_, 1.8 mM KH_2_PO_4_, pH 7.4).

The plates were washed three times with PBS and blocked with 400 µL of PBS-BSA 3% for 1 h at 37 °C. After ten PBS washes, 50 µL of the phage-antibody library was added to each well, and the plates were incubated at 37 °C for 2 h. Unbound phages were washed away using five washes with PBS-T (PBS containing 0.05% Tween20). Phage antibodies binding to ovomucoid and boiled egg white were eluted with 50 µL of 0.1 M glycine-HCl-1% BSA (pH 2.2) by scratching the well bottoms with a cut pipette tip for 10 min. Finally, the recovered phages were neutralized to pH 7 with Tris-Base 2 M (Sigma©, #ref T0440).

The eluted phage-antibodies were amplified by infecting a 2 mL culture of *E. coli* XL1-Blue grown in SB (10 g/mL of tetracycline) at an OD600 = 0.8–0.9. After 15 min of phage infection, a small aliquot was ten-fold diluted and plated to calculate the output titter. Following this, 6 mL of SB (supplemented with 20 µg/mL of carbenicillin and 10 µg/mL of tetracycline) was added and further incubated for 1 h at 37 °C (250 rpm). The carbenicillin concentration was raised to 50 µg/mL, and the culture was incubated in the same conditions for another 1 h. At this stage, 10^12^ VSCM13 helper phages were added. The culture volume was scaled up to 100 mL of SB (containing 50 µg/mL of carbenicillin and 10 µg/mL of tetracycline) and incubated for 2 h at 37 °C (300 rpm). Kanamycin was added to a concentration of 70 µg/mL, and the culture was incubated overnight at 37 °C (300 rpm). Phage-scFvs were precipitated with 4% (*w*/*v*) PEG8000 and 3% (*w*/*v*) sodium chloride. These amplified phage populations from the initial round of panning were tittered and used as input for the second round of selection against Gal d 1 and boiled egg white. A total of four rounds of selection were carried out following the protocol described above but modulating both the concentration of antigen and the number of washes in order to favor the selection of candidates with the highest affinity for the antigen (Table S2).

### 2.7. Characterization of the Ovomucoid-Binding Phage-scFvs

A total of 41 single colonies were randomly isolated from the output plates after the third and fourth rounds of biopanning for both selection strategies. These colonies were grown in 4 mL of SB medium (50 µg/mL of carbenicillin) and incubated at 37 °C until reaching an OD600 of 0.9–1. Subsequently, 10^12^ pfu/mL of VSCM13 helper phage preparation was added to the culture and further incubated at 37 °C with shaking for 2 h. Upon helper infection, kanamycin was added at a concentration of 70 µg/mL, leading to the production of phage-scFvs in the supernatant at 37 °C for 16 h. Selection of phage-antibody candidates with binding capacity to ovomucoid and egg white was performed using indirect ELISA as described in Section 2.8, employing the supernatants (diluted 1:1 in PBS) instead of the PEG-purified phages. Once positive phage-antibodies were identified and sequenced, an upscale production (100 mL of SB-carbenicillin medium) was carried out with the ovomucoid-binding individual clones using the same protocol. The phage-scFvs were purified from the supernatant using the PEG-NaCl protocol, as described in Section 2.6. and the resulting phage preparation was tittered using the top-agar methodology.

### 2.8. Indirect Phage-scFv ELISA

Microtiter plates (F96 MaxiSorp Nunc immunoplates, Thermo Fisher) were coated with 100 µL of extracts prepared at the concentrations indicated in each experiment and incubated for 1 h at 37 °C. After washing the plates 10 times with PBS, a blocking step with 200 µL of 1% BSA in PBS was performed at 37 °C for 1 h. Following another round of 10 washes, 100 µL of the diluted phages (10^9^ pfu/mL) were added to the wells, and plates were incubated for 1 h at room temperature. Unbound phage particles were removed using 10 PBS washes, and bound phages were detected by adding 100 µL of the anti-protein-VIII HRP-conjugated monoclonal mouse antibody (Sinobiological©, Beijing, China, #ref 11973) diluted 1:5000 in blocking buffer. The plate was incubated for 1 h at 37 °C, and PBS washed 10 times. Finally, the reaction was developed with 100 µL of TMB (Tetramethylbenzidine, Sigma©, #ref T0440) for 20 min in the dark and stopped with 50 µL of 1 M sulphuric acid. Absorbance measurements were performed at 450 nm using a FluoStar Optima™ spectrophotometer (BMG labtech©, Ortenberg, Germany).

To validate the results of this phage-scFv ELISA, the food products selected for this study were also analyzed with the commercial sandwich ELISA “Egg Assay Kit” from BioKits (Neogen, Lansing, MI, USA, #ref 902072T) following the manufacturer’s instructions. This assay used as a reference is based on a polyclonal antibody that also detects ovomucoid.

### 2.9. In Silico Modeling of SR-G1 scFv Structure and Its Interaction with Ovomucoid

The computational characterization of the structure of the leading scFv candidate (SR-G1) and its interaction with the antigen was undertaken to gain a more comprehensive understanding of the antibody. The SR-G1 DNA sequence was uploaded into IMGT/V-QUEST (https://www.imgt.org/IMGT_vquest/input, accessed on 17 February 2023), an alignment-oriented tool designed for the classification and categorization of antibody domains [28]. The predictive modeling of the scFv structure was performed using Abodybuilder2 (https://opig.stats.ox.ac.uk/webapps/sabdab-sabpred/sabpred/abodybuilder2/, accessed on 17 February 2023) [29]. Based on the obtained information, the antigen-antibody interaction was simulated using the HADDOCK 2.4 server (https://wenmr.science.uu.nl/haddock2.4/, accessed on 17 February 2023) [30,31]. Finally, the binding affinity (ΔG) of the complex and other parameters were calculated using PRODIGY (PROtein binDIng enerGY prediction) (https://wenmr.science.uu.nl/prodigy/, accessed on 17 February 2023) [32]. The resultant models were visualized using the ChimeraX software [33].

## 3. Results and Discussion

### 3.1. Construction of the Immune scFv Library

The rabbit spleen was selected as the source of antibodies for the construction of the ovomucoid-immune library due to its unique attributes as a secondary lymphoid organ. Within the spleen, B cells undergo a crucial affinity maturation process, leading to the selection of antibodies with higher affinity after exposure to the antigen [34]. This particular approach consistently yields a vast array of high-affinity binders specifically tailored to the target antigen [16].

**Figure 1:**
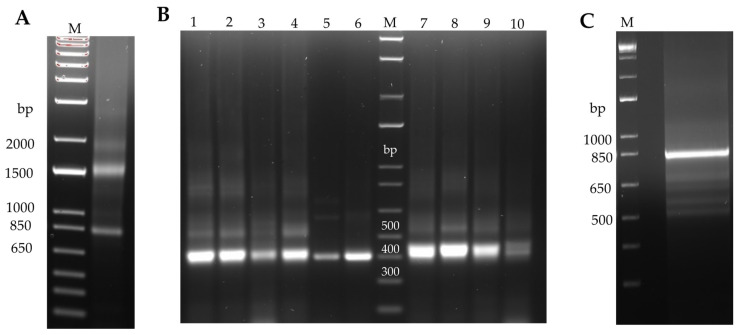
Construction of the rabbit phage display scFv library. (**A**) Total RNA extracted from the ovomucoid immunized rabbit spleen. (**B**) Representative amplicons that were obtained for the light chain (VL) gene repertoire of approximately 400 bp (lanes 1–6) and the heavy chain (VH) gene repertoire of approximately 415 bp (lanes 7–10). (**C**) Full-length scFv fragment assembled by SOE-PCR (~800 bp). M: DNA marker.

Figure 1A shows the 28S and 18S rRNA bands isolated from the ovomucoid-immunized rabbit spleen, which exhibited good integrity to be subsequently subjected to cDNA synthesis using general oligo(dT)18 primers. The obtained cDNA served as a template for the amplification of V_L_ and V_H_ genes using all possible combinations within a primer set (Table S1). A representative selection of the resulting V_L_ and V_H_ amplicons can be seen in Figure 1B. Gel-purified amplicons were mixed into two poles (light chains and heavy chains) to create all possible gene combinations and used to generate full-length scFvs using SOE-PCR, yielding the expected construct of approximately 800 bp (Figure 1C). The process of library construction by cloning the scFv sequences into the pComb3X vector and electroporation in *E. coli* XL-1 Blue cells led to the generation of an ovomucoid-immunized rabbit library with a repertoire size (RZ) of 3 × 10^6^. To verify the correct construction of the library, 20 random colonies were subjected to colony PCR and phagemid sequencing. All transformants were checked to display the scFv gene of the correct size via agarose gel electrophoresis, and DNA sequence analysis revealed abundant diversity at the complementary determining regions (CDRs) of the VL (CDR1−3 VL) and VH (CDR1−3 VH) chains of the assayed clones (Figure S1).

### 3.2. Biopanning of Anti-Ovomucoid Phage-scFv

Biopanning is an affinity selection technique for the selection of antibodies against a desired target from an antibody phage library. In this study, two biopanning strategies were designed to increase the possibilities of obtaining ovomucoid-binding scFvs from the constructed library that could recognize different epitopes of the antigen. In the first strategy, purified chicken ovomucoid was used as the target molecule coated in the microplate wells (OM-panning), while in the second strategy, the immobilized antigen consisted of boiled hen’s egg white extract (EW-panning). In both cases, four rounds of biopanning were performed, gradually increasing the selection stringency by adding additional washing steps while reducing the concentration of coated antigen (Table S2). The enrichment process of the phage display library was tracked by tittering the output phages after each of the four rounds of selection, which corresponds to the eluted phage-antibodies after antigen exposition used for *E. coli* infection and amplification (Figure S2). The results revealed that the number of phage particles recovered from the OM-panning increased 7-fold between the second and third rounds of selection, whilst this increase for the EW-panning was 12-fold. Such increment in phage recovery suggests that, for both strategies, phage variants targeting the desired molecule were likely selected after the third round of biopanning.

### 3.3. Polyclonal Phage-ELISA

**Figure 2:**
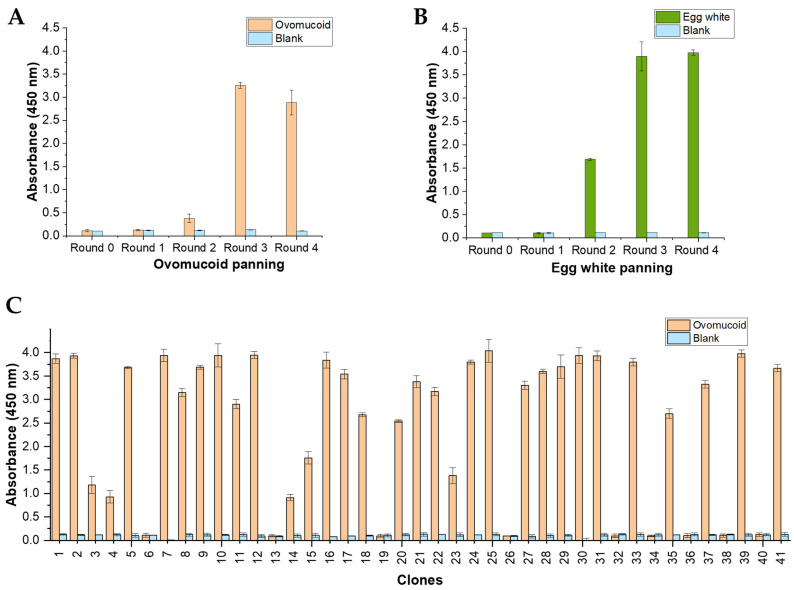
Characterization of the two biopanning strategies employing ovomucoid and boiled egg white as antigens for the selection of binding phage-scFvs. (**A**,**B**) Polyclonal indirect phage-ELISA of the recovered phage repertoires after each round of panning against (**A**) ovomucoid (OM) and (**B**) boiled egg white (EW). (**C**) Monoclonal phage-ELISA of the 41 individual clones isolated after the selection process (1–11 clones from the OM-panning and 12–41 from the EW-panning). The coating concentration used for ovomucoid was 10 µg/mL and 140 µg/mL for the egg white extract. The data are expressed as an average of duplicate measurements with their standard deviations.

To verify the enrichment of the library in ovomucoid-binding variants, an indirect polyclonal phage-ELISA with the phage populations amplified after each round of selection was conducted (Figure 2A,B). As can be observed, the output phages of the second round of selection showed a slight increase in ovomucoid recognition for both biopanning strategies. Ovomucoid detection was maximized when using the phage-scFv repertoires recovered after the third and fourth panning rounds. Remarkably, minimal cross-reactivity was observed against the BSA blocking solution (used as negative control) when using the phages recovered after enrichment of the constructed library. This finding suggests that there was no non-specific selection of phage variants against other components of the biopanning system. Thus, the ovomucoid-binding phage population was significantly enriched by the third round of selection in both biopanning strategies.

### 3.4. Screening of Anti-Ovomucoid Binders by Monoclonal Phage-ELISA

A total of 41 clones were randomly picked from the tittering plates of the last two selection rounds of each panning strategy (11 and 30 from the OM and EW panning, respectively). Induced phage supernatants were subjected to monoclonal phage-ELISA in immunoplates coated with ovomucoid (Figure 2C) and boiled egg white extract (results not shown due to their high similarity to ovomucoid recognition). A total of 31 out of 41 clones (75%) displayed high affinity to both ovomucoid and egg white extract. This substantial number of positive clones correlates with the findings observed in the polyclonal phage-ELISA, demonstrating that the immune phage library was largely enriched with phage particles exhibiting high affinity for the target antigen.

### 3.5. Sequence Analysis of the Positive Clones

Among the 31 sequenced ovomucoid-binding scFvs, only four revealed distinct amino acid sequences identified from the study of their complementary determining regions (CDRs), which are highly diverse regions responsible for the antibody’s specificity and antigen-binding site. Positive clones recovered from the OM-panning were identified as a unique clone named SR-G1. However, from the study of the EW-panning clones, four candidates with different frequencies of occurrence were obtained: SR-G1 at 54%, SR-G2 at 36%, SR-G3 at 5%, and SR-G4 at 5%. The presence of the same clone (SR-G1) in both strategies could be of great interest since this scFv-phage could detect the ovomucoid protein both in its native and purified form. In this sense, it could be possible that this clone was directed against epitopes with high heat resistance of ovomucoid, allowing the recognition of the protein even in cooked egg white since heat stability of ovomucoid has been widely proven [6].

The diversity of the primary antibody repertoire is mainly generated using the rearrangement of V, D, J (heavy chain), and V, J (light chain) genes. However, the pool of these genes in rabbits is more limited compared to other species [21]. To compensate for this limitation, the rabbit immune system employs both gene conversion and somatic hypermutation processes to further diversify the antibody repertoire [35]. The re-arranged genes of the four scFv clones isolated in this study, as elucidated using the IMGT^®^/V-Quest tool, are detailed in Table 2. Regarding the heavy chains, all the antibodies presented the same V gene (IGV1S69), which interestingly has been consistently reported in previous studies as the dominant gene in the VH repertoire of immunized rabbits but with a much smaller contribution in naïve rabbit libraries [20,36]. For J and D gene usage in the VH chains, both SR-G3 and SR-G4 clones used the IGHJ4 and IGHD7 genes, with the J gene being the most prevalent in rabbits (approximately 80%). On the other hand, the SR-G1 and SR-G2 clones utilized the considerably less common IGHJ6 gene (8%) [37] and also shared the same D gene (IGHD2). With respect to light chains, there was an overall prevalence of kappa chains, and each scFv belonged to different clonotypes (kappa V genes varied among all clones, and J genes were only shared in SR-G3 and SR-G4 clones). These results are in accordance with other studies that have shown lower bias in the VL repertoire, with no single gene contributing more than 20% to the overall repertoire [36]. In conclusion, the phage-scFv clones rescued after the biopanning process exhibited a high diversity of light chains but very similar heavy chain germlines genes. This observation suggests that, while the VH repertoire of rabbit antibodies is more limited than the VL in terms of functional genes available for rearrangement, the heavy region has played a leading role in the selection of high-affinity clones against ovomucoid.

Commonly, the CDR3 regions of both VL and VH are the most hypervariable regions of antibodies, thus playing a critical role in antigen-specific recognition. The length of the VH-CDR3 of the selected clones was 13 ± 2 amino acids, slightly smaller than that of humans (15 ± 4 amino acids) [21,38]. However, the length of the VL-CDR3 (12 ± 1 amino acids) was significantly longer than that of its human and mouse counterparts (9 ± 1 amino acid) [39]. This difference in length might be attributed to the greater diversity of the VL repertoire in rabbits, which compensates for the limited number of functional germline VH genes [21].

### 3.6. Characterization of the Anti-Ovomucoid Phage-scFvs Clones

**Figure 3:**
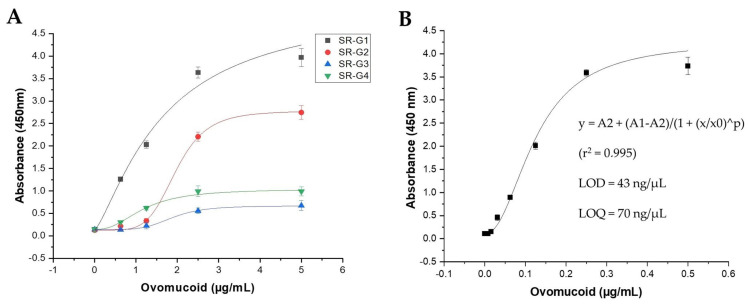
Characterization of ovomucoid-binding scFvs by phage-ELISA. (**A**) Comparative dose–response curve of the four phage-scFv candidates against ovomucoid (0.625–5 µg/mL). (**B**) Calibration curve using a logistic adjustment of the phage-ELISA with the clone SR-G1 performed with ovomucoid dilutions in PBS (0.008–0.5 µg/mL). Origin 2021 software was employed to plot and analyze the experimental data expressed as an average of triplicate measurements with their standard deviations.

An indirect phage-ELISA was implemented to determine the binding properties of the four isolated clones. The optimal concentration of phage-scFv as the primary antibody of the assay was determined to be 10^9^ phage particles per well. The dose-response curves were generated by analyzing increasing concentrations of ovomucoid (0.625–5 µg/mL) in PBS (Figure 3A). Although all the scFvs satisfactorily recognized the ovomucoid protein, the response of clone SR-G1 at low protein concentrations was considerably higher than the others. While slightly weaker, the SR-G2 clone also showed good recognition characteristics, suggesting that the high degree of similarity found between their VH regions (same clonotype) could indicate that both clones target the same ovomucoid epitope. In light of these results, subsequent characterization experiments were only focused on the SR-G1 phage-scFv.

The limits of detection (LOD) and quantitation (LOQ) of clone SR-G1 were determined from the ovomucoid concentrations of the dose–response curve that corresponded to the average absorbance of ten blank measurements plus three and ten times the standard deviation, respectively (Figure 3B). The calculated LOD of 43 ng/mL and LOQ of 79 ng/mL indicated the appropriate assay sensitivity for egg detection in foods, which is comparable to that of other commercial ELISAs used for this purpose [40].

Currently, there is no legislation on the minimum doses required to declare egg on the label of egg-containing foods. However, some studies suggest the necessity of a sensitivity threshold of 10 ppm for egg white detection in foods to ensure a 99% safety level for allergic patients [41]. Considering that ovomucoid represents 11% of egg white proteins, the phage-ELISA developed in this study would meet this requirement, presenting a limit of detection within the established limits. In this sense, phage display technology offers the opportunity to improve the performance of the assay using in vitro affinity maturation methodologies to find new clones with enhanced sensitivity derived from the SR-G1 antibody. To study the specificity of the SR-G1 phage-ELISA, extracts from different ingredients commonly used in food manufacturing were tested (Table 1). All the heterologous species analyzed developed absorbance signals lower than the LOD of the assay. Interestingly, egg whites from various poultry species were analyzed, demonstrating that SR-G1 phage-scFv recognized exclusively the hen’s egg white extract, reporting absorbance values below the LOD when egg whites from goose, duck, ostrich, or quail were examined. Additionally, cooked hen egg yolk, as well as other egg allergens (ovalbumin and lysozyme), were analyzed (Figure S3). The results indicated that the SR-G1 clone specifically targeted the egg white ovomucoid allergen since the slight response observed for ovalbumin and lysozyme could probably be attributed to the fact that these commercial reagents are not thoroughly purified and could probably present residual ovomucoid.

Other ELISA methods based on monoclonal antibodies (mAb) have been reported in the literature. Hirose et al. developed different mAb capable of recognizing native and heat-denatured ovomucoid, reporting limits of detection of 100 ng/mL in a sandwich ELISA based on the best candidate [42]. This test sensitivity was further enhanced 100-fold by using an oligoclonal cocktail of the mAbs [43]. Li et al. reported a sandwich ELISA with an LOD of 0.041 ng/mL; however, this test showed a low level of cross-reactivity with chicken serum [44]. Recently, Hwan-shin et al. described a phage virus-based electrochemical biosensor based on a cyclic peptide obtained by phage display that was able to detect 120 ng/mL of ovomucoid [45]. To the best of our knowledge, studies on the production of recombinant antibodies against ovomucoid have not been described to date, being the present study the first in this field. Furthermore, the sensitivity achieved with the phage-ELISA based on the scFv-SR-G1 was comparable or even superior to that described in previous studies.

### 3.7. Determination of Ovomucoid in Commercial Food Products

The applicability of the developed indirect phage ELISA for ovomucoid detection in foodstuffs was assessed using the analysis of 23 commercial products with different labels regarding the presence of eggs (Table 3). Further, all samples were analyzed in parallel with a commercial sandwich ELISA, also targeting ovomucoid protein, in order to validate the results. The samples were categorized into four groups according to their labeling: (A) products that declared egg as an ingredient (13 samples); (B) products that declared only egg yolk as an ingredient (3 samples); (C) products with precautionary egg labeling such as “may contain egg traces” (3 samples) and (D) products with no declaration regarding egg presence (5 samples).

The developed phage-ELISA successfully detected eggs in the food products that included eggs in their ingredient list. Of the three products that declared only egg yolk as an ingredient, all of them resulted positive with the sandwich-ELISA, while the phage-ELISA detected the presence of ovomucoid only in one of the samples. This discrepancy could be attributed to possible cross-reactivity of the polyclonal ELISA with egg proteins other than ovomucoid since ovomucoid is theoretically found in egg whites. Probably, the cabbage salad that yielded positive results in both ELISAs could be contaminated with traces of egg white. Regarding the samples with precautionary egg labeling, one product showed positive absorbance values when tested with the phage-ELISA and two products when tested with the sandwich ELISA. Lastly, among the 5 samples that did not declare egg, 4 of them resulted negative with both assays, demonstrating the good specificity of the developed assay. The other product, consisting of cold meat, yielded positive absorbance values in the phage-ELISA, a result that was also confirmed with the sandwich assay. Although the absorbance signal generated was relatively low, possibly indicating cross-contamination, such mislabeling of the product could pose a serious health risk to allergic consumers. In this regard, the SR-G1 antibody has demonstrated its potential to detect this mislabeling and could be implemented for use in allergen management by the food industry.

The discrepancies described between the two ELISA methods could be explained using the different sensitivities reported for both assays. In this regard, the LOD of the commercial ELISA was 10 ng/mL of ovomucoid, a four-fold lower LOD than that achieved with the phage-ELISA developed in this work. This could explain why samples with low concentrations of ovomucoid, as detected with the commercial kit, were undetected by the SR-G1 ELISA. Furthermore, it is important to consider that the accessibility of ovomucoid epitopes can be greatly influenced by the food matrix and food processing methods. Consequently, the SR-G1 scFv could be unable to recognize its specific epitope, whereas the polyclonal antibody has the potential to recognize a diverse range of different epitopes of the ovomucoid molecule.

### 3.8. Molecular Docking of the Ovomucoid-Binding scFv SR-G1

**Figure 4:**
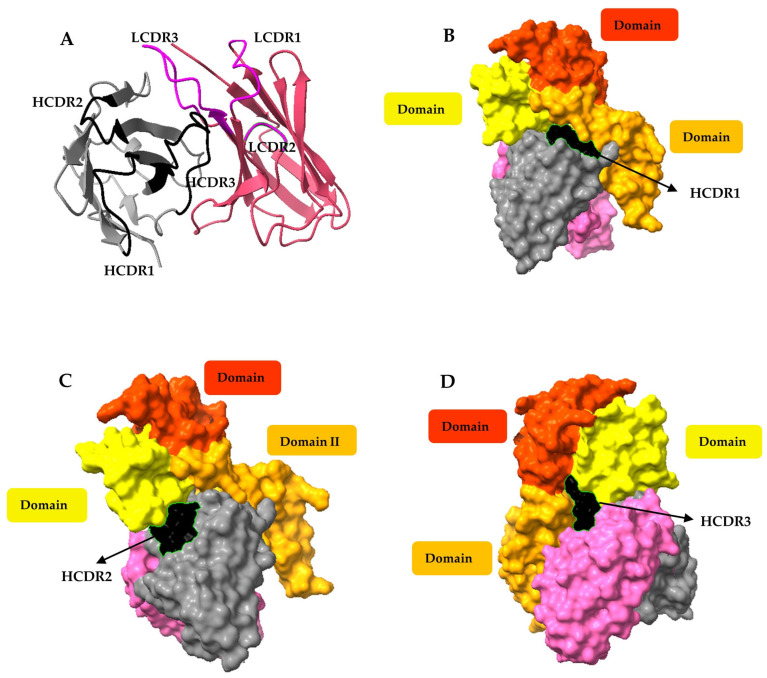
Computational models of the SR-G1 scFv structure and its antigen-antibody interaction. (**A**) SR-G1 structure prediction using ABodyBuilder2. The backbones of the heavy and light chains are shown in grey and pink, respectively, and the complementary determining regions indicated (LCDR1-3 and HCDR1-3). (**B**–**D**) Representation of the main interaction surfaces between the ovomucoid domains and the SR-G1 heavy CDRs using Haddock2.4.: (**B**) HCDR1, (**C**) HCDR2 and (**D**) HCDR3.

To gain deeper insight into the characteristics of the SR-G1 antibody fragment, an in silico approach for studying the molecular interaction between the scFv and hen’s ovo-mucoid was conducted. Firstly, based on the sequencing results of the SR-G1 clone, the 3D-structure prediction of the molecule was generated using AbodyBuilder2 (Figure 4A), a deep learning program that performs predictions of antibodies with high accuracy. Notably, this tool has shown exceptional precision in predicting the structure of the CDR-H3, an area where other methods have struggled to achieve accurate predictions [46].

While there are high-resolution structures published for the ovomucoid of various poultry species, no experimental data (such as X-ray crystallography or cryo-microscopy structures) have been elucidated for the hen’s egg ovomucoid. It should be noted that the ovomucoid protein varies in several amino acids among the different species, and these discrepancies in sequence could probably affect the epitopes of the allergens [47]. Therefore, since the SR-G1 scFv specifically detects hen egg ovomucoid, we decided to employ the alpha-fold-generated chicken ovomucoid model uploaded in the UNIPROT database as P01005 (or IOVO_CHICK). Molecular docking between the generated 3D structure of SR-G1 and ovomucoid was conducted using the HADDOCK 2.4 server. Since this tool employs a local docking method that requires specifying the active binding sites of the ligands, the most likely interaction regions were defined as the CDRs of the SR-G1 scFv and immunoglobulin-binding epitopes of the chicken ovomucoid. These regions were previously determined by Mine et al. from the reactivity of pooled sera from egg-allergic patients, in which 9 and 8 linear ovomucoid epitopes binding to IgE and IgG, respectively, were identified [48].

Multiple docking models were computed, and the one with the best parameters (HADDOCK score = −51.8 ± 7.5; RMSD = 0.5 ± 0.3; Van der Waals energy = −88.2 ± 6.0; Z-score = −1.4) was selected for further analysis. When selecting the best protein/ligand model, many studies only take into account the best HADDOCK score, considering that a more negative score indicates that the complex is energetically stable and the binding is favorable. However, the SR-G1/ovomucoid model was selected regarding the RMSD, where a score below 2 Å is considered to have high accuracy [49]. Therefore, although the selected model had a slightly worse HADDOCK score than other estimations, the significantly better RMSD was considered an important parameter in the docking analysis to avoid bias [50]. Based on the modeled complex, the PRODIGY tool was used to predict binding energy and identify the total number of interactions between the two molecules (charged-charged, charged-polar, charged-apolar, polar-polar, polar-apolar, and apolar-apolar interactions) as summarized in Table S3. Overall, the antibody SR-G1 was predicted to have more than 110 interactions with the hen’s ovomucoid protein.

As depicted in Figure 4B–D, the main identified paratopes were located in the heavy chain of the SR-G1 scFv. The HCDR1 (Figure 4B) of the SR-G1 scFv interacts with lysine and acidic glutamic residues (positions 154–155) that form part of an epitope located in the domain II of the ovomucoid protein. On the other hand, the HCDR2 predominantly interacts with the ovomucoid’s domain III (Figure 4C), and the HCDR3 forms a loop that fits between domains II and III (Figure 4D) thanks to its long size. Interestingly, the main interactions occur between the heavy chain of the scFv and the C-terminal region of the third domain of the ovomucoid, which has been described as the most immunogenic region of this egg allergen [10]. Although the contribution of the light chain to the interaction appears to be comparatively weaker, it might contribute to the overall stability of the binding complex. While these results proceed from simulations, the high quality of the docking procedure supports the conclusion that SR-G1 scFv is a promising ovomucoid binder, and all the information derived from the models is valuable data for further improvement in the antibody properties.

## 4. Conclusions

This work describes for the first time the generation of a recombinant antibody fragment intended for ovomucoid detection in foods using phage display technology. The recombinant scFv SR-G1, obtained from a rabbit immune library, was capable of binding specifically to the hen’s egg ovomucoid allergen. Based on this scFv, a phage-ELISA test was successfully developed, yielding a LOD of 43 ng/mL and demonstrating its applicability for detecting egg-diverse food products. Further, in silico modeling revealed a close contact between the scFv and the third domain of chicken ovomucoid, suggesting a potential location for the allergen epitope recognized by the antibody. The SR-G1 scFv derived from this study could serve as a novel ovomucoid detection probe in order to improve food safety egg allergen management and ensure regulatory compliance in the food industry.

## Figures and Tables

**Table 1:** Heterologous species that were included in the specificity assays.

**Vegetal Species**		
Almond (*Prunus dulcis*)	Kiwifruit (*Actinidia deliciosa*)	Rice (*Oryza sativa*)
Apple (*Malus domestica*)	Maize (*Zea mays*)	Sesame (*Sesamum indicum*)
Banana (*Musa acuminate*)	Mandarin orange (*Citrus reticulate*)	Spinach (*Spinacea oleracea*)
Carrot (*Daucus carota*)	Oats (*Avena sativa*)	Soy (*Glycine max*)
Cashew nut (*Anacardium occidentale*)	Onion (*Allium cepa*)	Tomato (*Solanum lycopersicum*)
Chia (*Salvia hispanica*)	Pear (*Pyrus communis*)	Walnut (*Juglans regia*)
Chickpea (*Cicer arietinum*)	Poppy seed (*Papaver rhoeas*)	Wheat (*Triticum aestivum*)
Flaxseed (*Linum usitatissimum*)	Quinoa (*Chenopodium quinoa*)	
Garlic (*Allium sativum*)	Red peppers (*Capsicum annuum*)	
Hazelnut (*Corylus avellana*)		
**Animal species**		
Beef (*Bos taurus*)	Pork (*Sus scrofa domestica*)	
Chicken (*Gallus gallus domesticus*)	Turkey (*Meleagris gallopavo*)	
**Others**		
Milk (*Bos taurus*)	Ostrich’s egg (*Struthio camelus*)	Quail’s egg (*Coturnix coturnix*)
Hen’s egg (*Gallus gallus domesticus*)	Duck’s egg (*Anas platyrhynchos domesticus*)	Goose’s egg (*Anas pedes sulfurate*)

**Table 2:** Identification of the re-arranged genes coding the light and heavy chains of the anti-ovomucoid scFvs. The VJ and VDJ genes were elucidated using the IMGT^®^/V-Quest tool.

Clone	Light Chain	Heavy Chain
SR-G1	IGKV1S36*01	IGHV1S69*01
	IGKJ1-2*04	IGHJ6*02
		IGHD2-1*01
SR-G2	IGKV1S34*01	IGHV1S69*01
	IGKJ1-2*01	IGHJ6*02
		IGHD2-1*01
SR-G3	IGKV1S44*01	IGHV1S69*01
	IGKJ1-2*02	IGHJ4*02
		IGHD7-1*01
SR-G4	IGKV1S4*01	IGHV1S69*01
	IGKJ1-2*02	IGHJ4*02
		IGHD7-1*01

**Table 3:** Comparison in the detection of eggs in commercial food products using the SR-G1 phage-ELISA developed in this work and a polyclonal ELISA kit commercially available.

Label Statement	Product	Number of Samples Analysed	Phage-ELISA	Polyclonal ELISA
Egg declared as ingredie+nt	Biscuits	1	+(1)	+(1)
	Cake	1	+(1)	+(1)
	Flan	2	+(2)	+(2)
	Meat product	3	+(3)	+(3)
	Omelet	2	+(2)	+(2)
	Sandwich	1	+(1)	+(1)
	Salad	2	+(2)	+(2)
Egg yolk declared as ingredient	Bakery	1	−(1)	+(1)
	Salad	2	+(1)/−(1)	+(2)
May contain egg traces	Meat product	3	+(1)/(−2)	+(2)/(−1)
Not declaring to contain egg or traces	Breakfast cereal	1	−(1)	−(1)
	Seeds	1	−(1)	−(1)
	Snack	1	−(1)	−(1)
	Meat	2	+(1)/−(1)	+(1)/−(1)

## Data Availability

Data is contained within the article.

## Supplementary Materials

The following supporting information can be downloaded at: https://www.mdpi.com/article/10.3390/foods12203831/s1, Table S1: Oligonucleotides used for the amplification, assembly, and sequencing of the immune phage library; Table S2: Conditions employed in the biopanning process against ovomucoid and boiled egg white throughout the four rounds of selection; Table S3: PRODIGY results generated from the interactions of SR-G1 and hen’s egg ovomucoid molecule; Figure S1: Analysis of scFv genes diversity of 20 single colonies randomly selected from the constructed immune rabbit phage display scFv library; Figure S2: Evaluation of the library enrichment by phage titration after each round of selection for the ovomucoid and boiled egg white panning strategies; Figure S3: Specificity of the SR-G1 phage-ELISA against egg white (140 µg/mL), egg yolk (140 µg/mL) and major allergenic hen’s egg proteins at a concentration of 10 µg/mL.
