# Supplementary material for: Generation of an Ovomucoid-Immune scFv Library for the Development of Novel Immunoassays in Hen’s Egg Detection
Source: Foods. 2023 Oct 19;12(20):3831. doi: 10.3390/foods12203831 (PMC10606182; doi:10.3390/foods12203831)
Supplement: Supplementary file 1 [file foods-12-03831-s001.zip › foods-2665200-supplementary.pdf]

Table S1. Oligonucleotides used for the amplification, assembly and sequencing of the immune phage library.

**Primer name**	**Sequence**
V_L forward primers (4)	
VL5′ *Sfi*I- K	5′ GGGCCCAGGCGGCCGAGCTCGTGMTGACCCAGACTCCA 3′
	5′ GGGCCCAGGCGGCCGAGCTCGATMTGACCCAGACTCCA 3′
	5′ GGGCCCAGGCGGCCGAGCTCGTGATGACCCAGACTGAA 3′
VL5′ *Sfi*I- λ	3′ GGGCCCAGGCGGCCGACTCAGTCGCCCTC 5′
V_H reverse primers (4)	
VL3′ Linker- K	5′ GGAAGATCTAGAGGAACCACCCCCACCACCGCCCGAGCCACCGCCACCAGAGGATTTGATTTCCACATTGGTGCC 3′
	5′ GGAAGATCTAGAGGAACCACCCCCACCACCGCCCGAGCCACCGCCACCAGAGGATAGGATCTCCAGCTCGGTCCC 3′
	5′ GGAAGATCTAGAGGAACCACCCCCACCACCGCCCGAGCCACCGCCACCAGAGGATTTGACSACCACCTCGGTCCC 3′
VL3′ Linker- λ	5′ GAAGATCTAGAGGAACCACCCCCACCACCGCCCGAGCCACCGCCACCAGAGGAGCCTGTGACGGTCAGGGTCCC 3′
V_H forward primers (4)	
VH5′ Linker	5′ GGTGGTTCCTCTAGATCTTCCCAGTCGGTGGAGGAGTCCRGG 3′
	5′ GGTGGTTCCTCTAGATCTTCCCAGTCGGTGAAGGAGTCCGAG 3′
	5′ GGTGGTTCCTCTAGATCTTCCCAGTCGYTGGAGGAGTCCGGG 3′
	5′ GGTGGTTCCTCTAGATCTTCCCAGSAGCAGCTGRTGGAGTCCGG 3′
V_H reverse primers (1)	
VH3′ *Sfi*I	5′ CCTGGCCGGCCTGGCCACTAGTGACTGAYGGAGCCTTAGGTTGCCC3′
Overlap extension PCR primers	
5′SfiI-VL	5′ GAGGAGGAGGAGGAGGAGGCGGGCCCAGGCGGCCGAGCTC 3′
3′SfiI-VH	5′ GAGGAGGAGGAGGAGGAGCCTGGCCGGCCTGGCCACTAGTG 3′
Sequencing primers	
ompAseq	5′ AAGACAGCTATCGCGATTGCAG 3′
g-back	5′ GCCCCCTTATTAGCGTTTGCCATC 3′

Table S2. Conditions employed in the biopanning process against ovomucoid and boiled egg white throughout the four rounds of selection.

Biopanning round	Coated ovomucoid (μg/mL)	Coated boiled egg white (μg/mL)	Number of washes
1	10	100	5
2	10	100	10
3	5	50	15
4	5	50	15

Table S3. PRODIGY results generated from the interactions of SR-G1 and hen's egg ovomucoid molecule.

ΔG (kcal/mol)	K_d (nM) at 25 °C	ICs charged-charged	ICs charged-polar	ICs charged-apolar	ICs polar-polar	ICs polar-apolar	ICs apolar-apolar	NIS charged	NIS apolar
-12.3	0.93	15	24	30	13	21	8	22.65	38.24

Abbreviations used: ΔG: binding affinity as Gibbs free energy; K_d: binding affinity as dissociation constant; ICs: number of interatomic contacts; NIS: non-interacting surfaces.

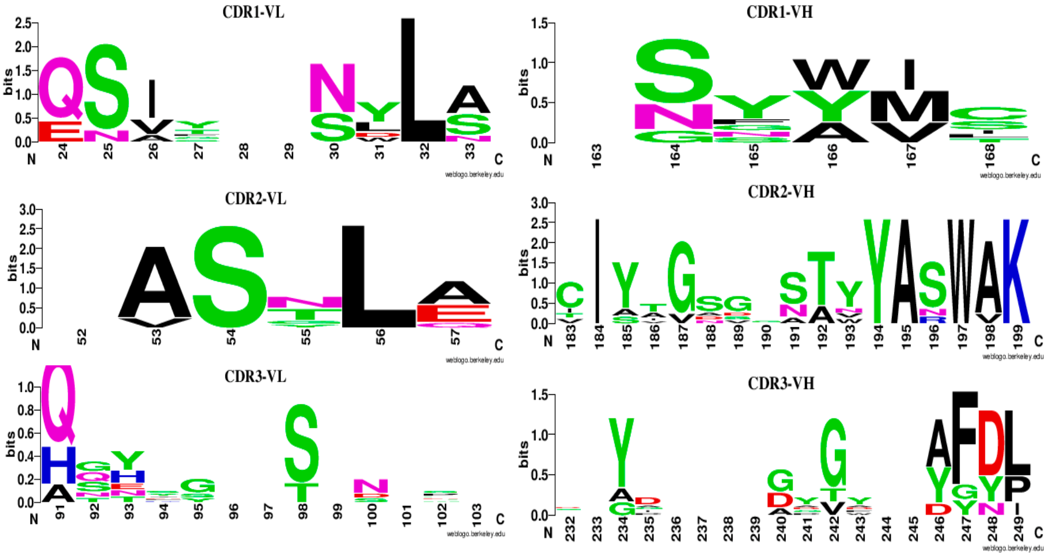

Figure S1. Analysis of scFv genes diversity of 20 single colonies randomly selected from the constructed immune rabbit phage display scFv library. The plots were generated by WebLogo website (http://weblogo.berkeley.edu/logo.cgi) and illustrate the generated diversity of the complementarity determining regions (CDRs) within the variable regions of both light (CDR1-3 VL) and heavy chains (CDR1-3 VH) of the scFv sequences.

.

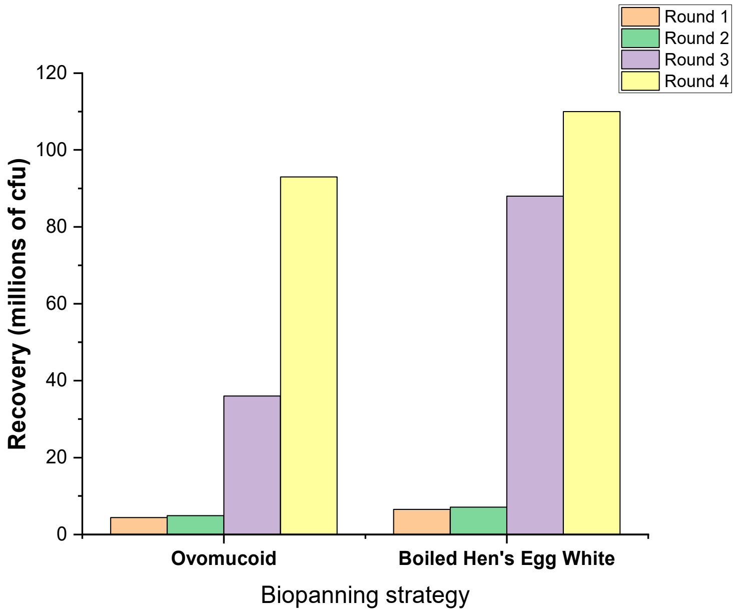

Figure S2. Evaluation of the library enrichment by phage titration after each round of selection for the ovomucoid and boiled egg white panning strategies.

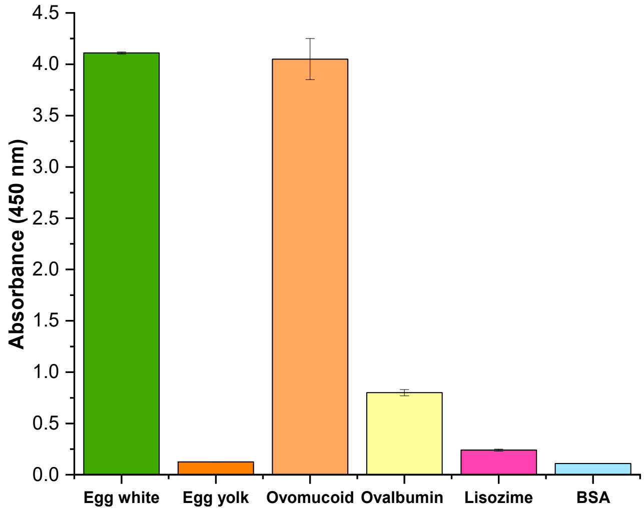

Figure S3. Specificity of the SR-G1 phage-ELISA against egg white (140 µg/mL), egg yolk (140 µg/mL) and major allergenic hen's egg proteins at a concentration of 10 µg/mL. The data is expressed as an average of duplicate measurements with their standard deviations.
